# Supplementary material for: Cooperative STAT/NF-κB signaling regulates lymphoma metabolic reprogramming and aberrant GOT2 expression
Source: Nat Commun. 2018 Apr 17;9:1514. doi: 10.1038/s41467-018-03803-x (PMC5904148; doi:10.1038/s41467-018-03803-x)
Supplement: Supplementary file 1 — Supplementary Information [file 41467_2018_3803_MOESM1_ESM.pdf]

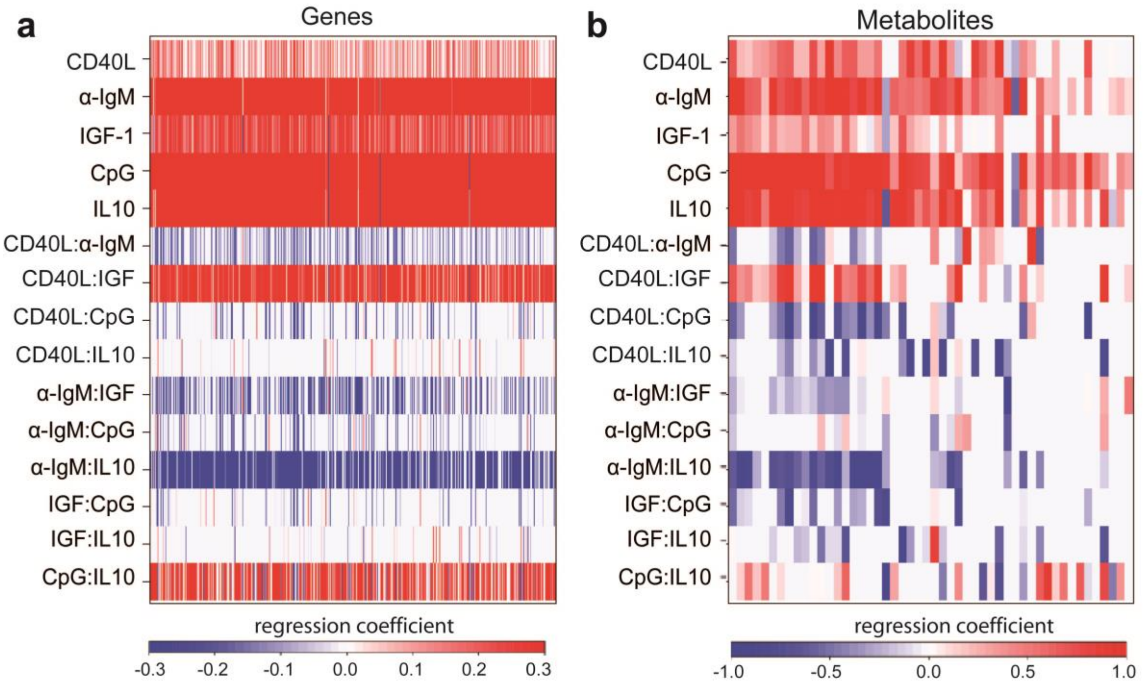

Supplementary Figure 1: Combined IL10+CpG stimulation of P493-6 MYC^{low} cells synergistically induces gene expression and metabolic changes. P493-6 cells were stimulated as described before [13]. The effects of single and dual stimuli on (a) global gene expression and (b) intracellular metabolite concentrations were estimated by regularized linear regression. The regression coefficients are given in the heatmaps. Single stimuli increased the expression of the majority of genes. Administration of all possible pairwise combinations of stimuli induced synergistic effects on gene expression and metabolite abundance, evident from the interaction terms of the linear model (*e.g.*, the coefficient CpG:IL10 representing the additional change in gene expression or metabolite abundance beyond the sum of the coefficients of IL10 and CpG). For 81% of the gene models, the Lasso selected the term of the interaction between IL10 and CpG, indicating its global importance when explaining gene expression changes by the environmental stimuli.

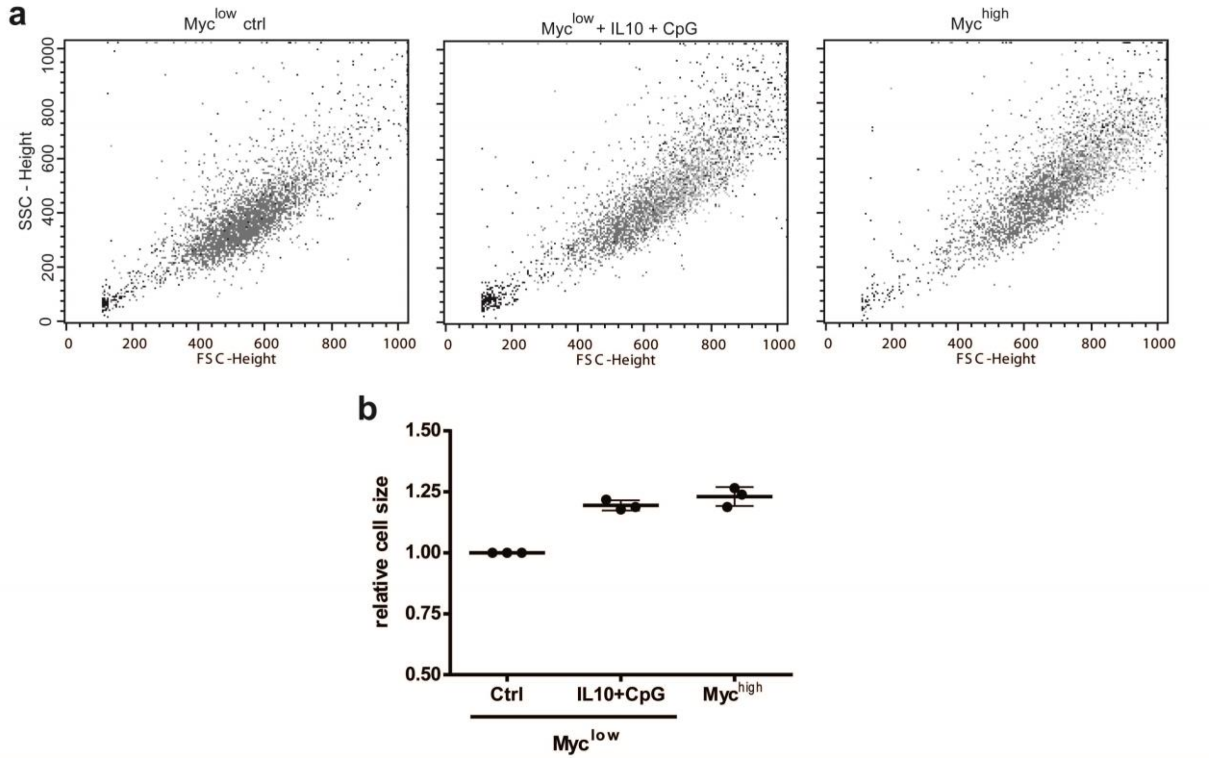

Supplementary Figure 2: P493-6 MYC^{low} cells show increased cell size upon IL10+CpG stimulation. (a) Forward/sideward scatter plots of flow cytometric measurements of unstimulated P493-6 MYC^{low}, IL10+CpG stimulated P493-6 MYC^{low} and P493-6 MYC^{high} cells, respectively. (**b**) Graphical presentation of the relative cell size of unstimulated P493-6 MYC^{low}, IL10+CpG stimulated P493-6 MYC^{low} and P493-6 MYC^{high} cells. Error bars represent mean±SD.

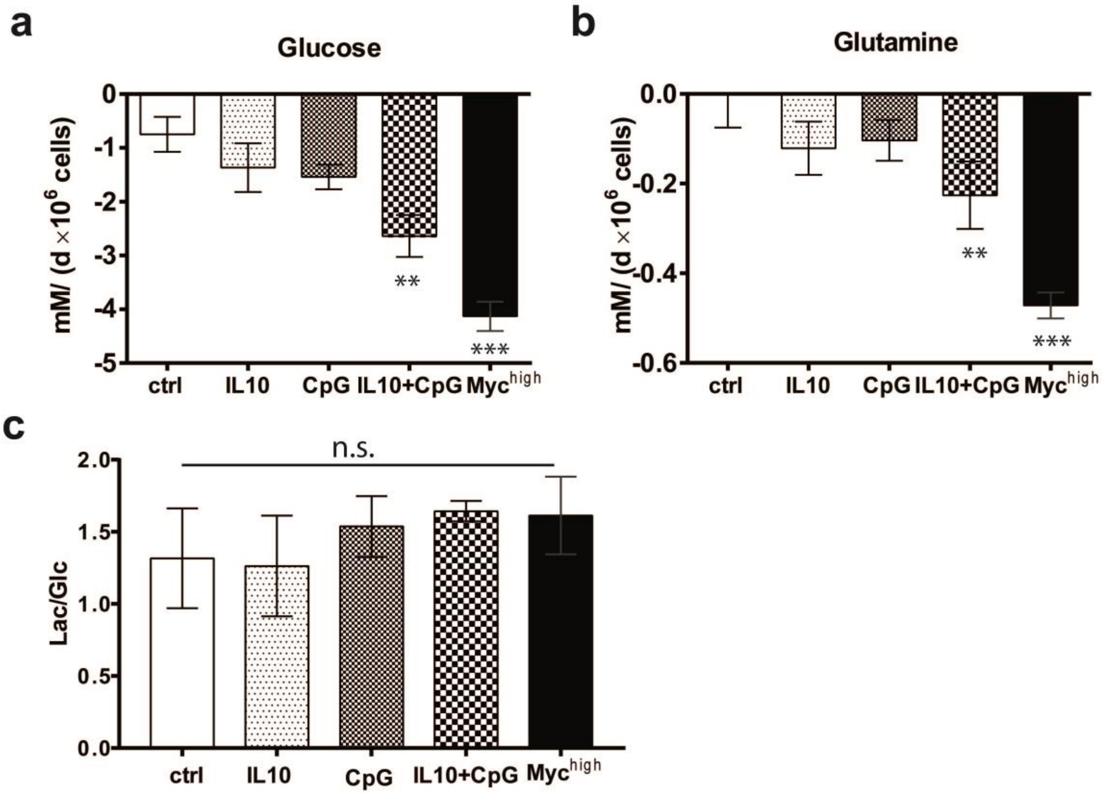

Supplementary Figure 3: IL10+CpG stimulation increases glucose and glutamine consumption of P493-6 MYC^{low} cells without significantly affecting the lactate-to-glucose molar ratio. Consumption of (**a**) Glc and (**b**) Gln by control, IL10-, CpG- and IL10+CpG-stimulated P493-6 MYC^{low} and unstimulated P493-6 MYC^{high} cells as measured by NMR spectroscopy. (**c**) The lactate-to-glucose molar ratio. Mean±SD of three independent experiments are given. Results from Bonferroni post hoc tests on a one-way ANOVA relative to Myc^{low} ctrl are presented (***p<0.001, ***p<0.01).

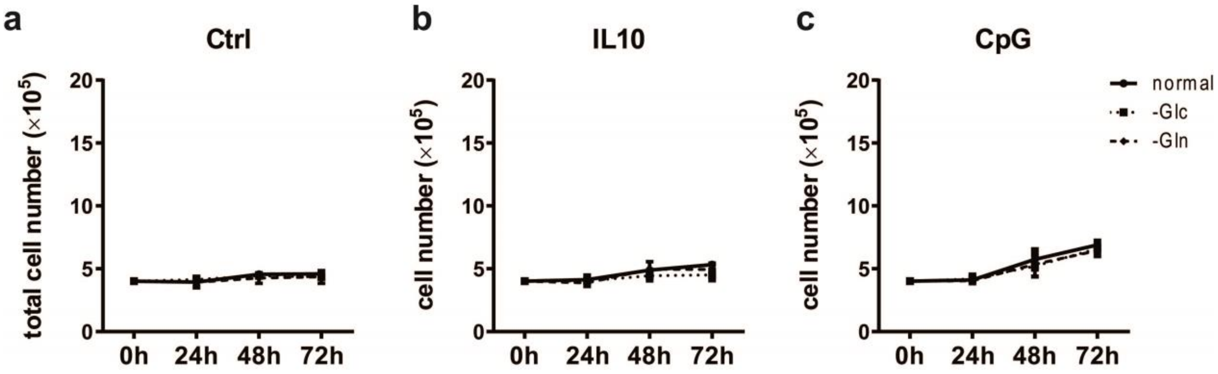

Supplementary Figure 4: Glutamine deprivation does not affect unstimulated or single stimulated MYC^{low} cells. Relative cell counts of unstimulated (**a**), IL10- (**b**) or CpG- (**c**) stimulated P493-6 MYC^{low} cells grown in media without glucose (-Glc) or glutamine (-Gln). Total cell count was measured every 24h. Mean±SD of three independent experiments are depicted.

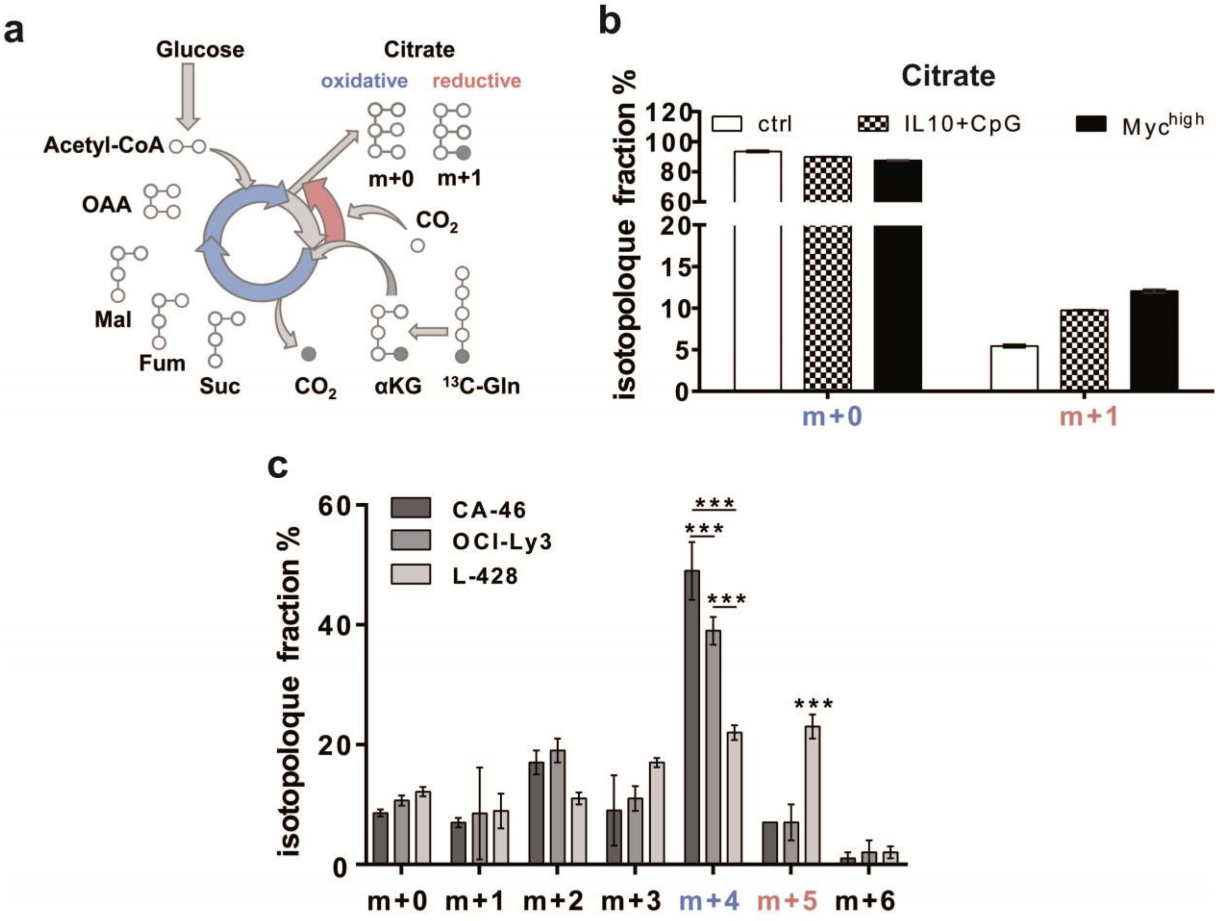

Supplementary Figure 5: ^{13}C-Isotopologue distribution of citric acid. (**a**) Scheme of oxidative decarboxylation (blue) and reductive carboxylation (red). (**b**) Isotopologue distribution of citric acid in unstimulated and IL10+CpG-stimulated P493-6 MYC^{low} and unstimulated P493-6 MYC^{high} cells treated with 1-^{13}C-glutamine for 24 hours. (**c**) Isotopologue distribution of citric acid in CA-46, OCI-Ly3 and L428 lymphoma cell lines treated with $^{13}C_5$-glutamine for 24 hours. Please refer also to Figure 2B for the scheme of oxidative decarboxylation. All values are given as mean±SD of three independent replicates. Results from Bonferroni post hoc tests on a two-way ANOVA are presented (***p<0.001).

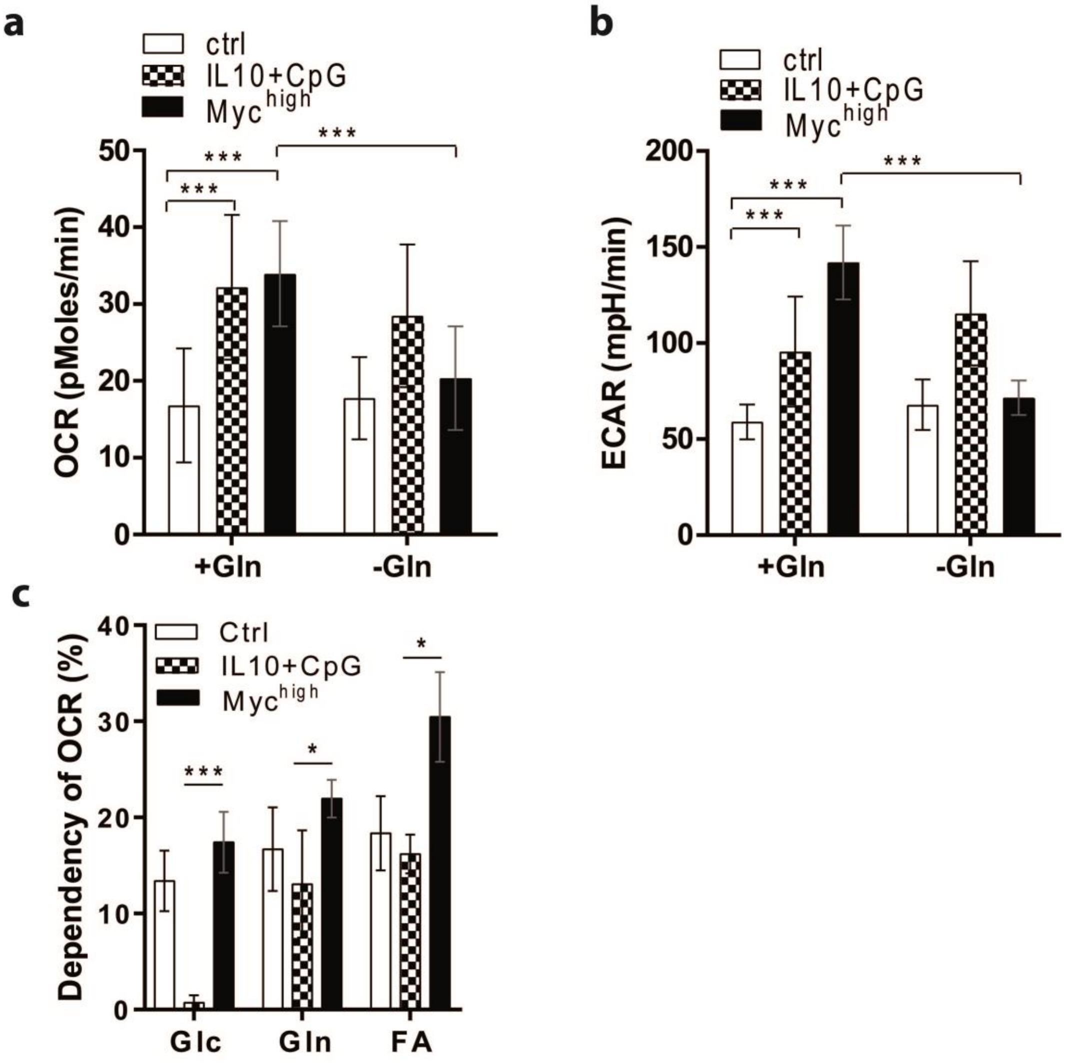

Supplementary Figure 6: Oxygen consumption and extracellular acidification rates of IL10+CpG-stimulated P493-6 MYC^{low} cells are less dependent on glutamine than those of P493-6 MYC^{high} cells.

(**a**) Basal OCR of IL10+CpG-stimulated P493-6 MYC^{low} and unstimulated P493-6 MYC^{high} cells under different media conditions. Cells were seeded in media with or without Gln and OCR was measured 24h after seeding. (**b**) ECAR of samples shown in A. (**c**) Basal OCR of IL10+CpG-stimulated P493-6 MYC^{low} and unstimulated P493-6 MYC^{high} cells after inhibition of mitochondrial pyruvate carrier (MPC1) by UK5099, glutaminase by BPTES, and carnitine palmitoyltransferase 1 (CPT1) by etomoxir. Mean±SD of three independent experiments and results from Bonferroni post hoc tests on a one-way ANOVA are presented (*p<0.05, **p<0.01, ***p<0.001).

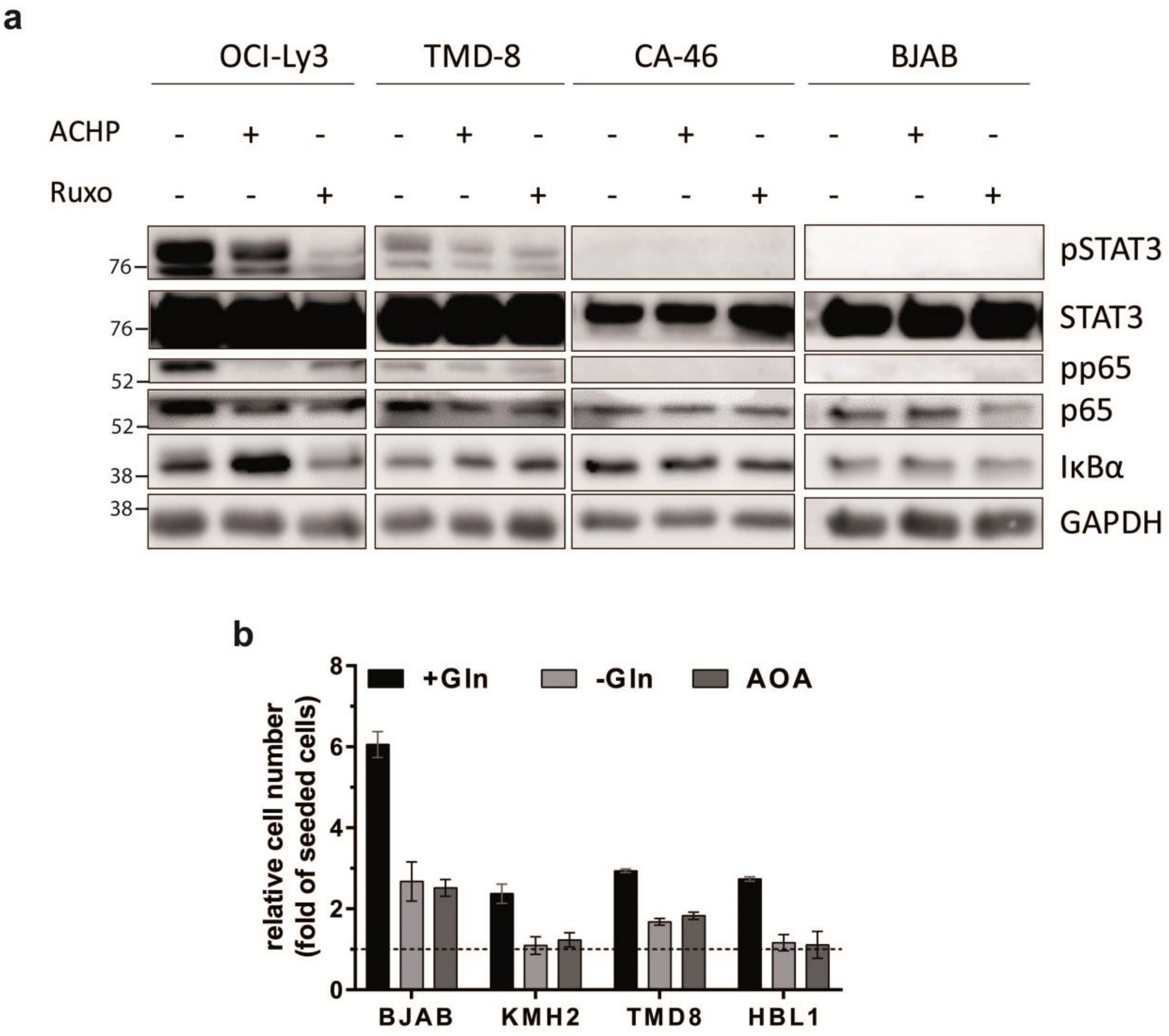

Supplementary Figure 7: NF-κB and STAT3 activation in different lymphoma cell lines and the effect of Gln deprivation or AOA treatment on cell proliferation. (**a**) Immunoblots of STAT3 and p65 phosphorylation and IkBα expression in BJAB, CA-46, OCI-Ly3, and TMD8 cells, respectively, with and without ACHP or ruxolitnib treatment as described in **Figure 6**. (**b**) Changes in relative cell count of the B lymphoma cell lines BJAB, KM-H2, HBL1, and TMD8 after glutamine deprivation and AOA treatment are shown. Mean±SD of three independent experiments are given.

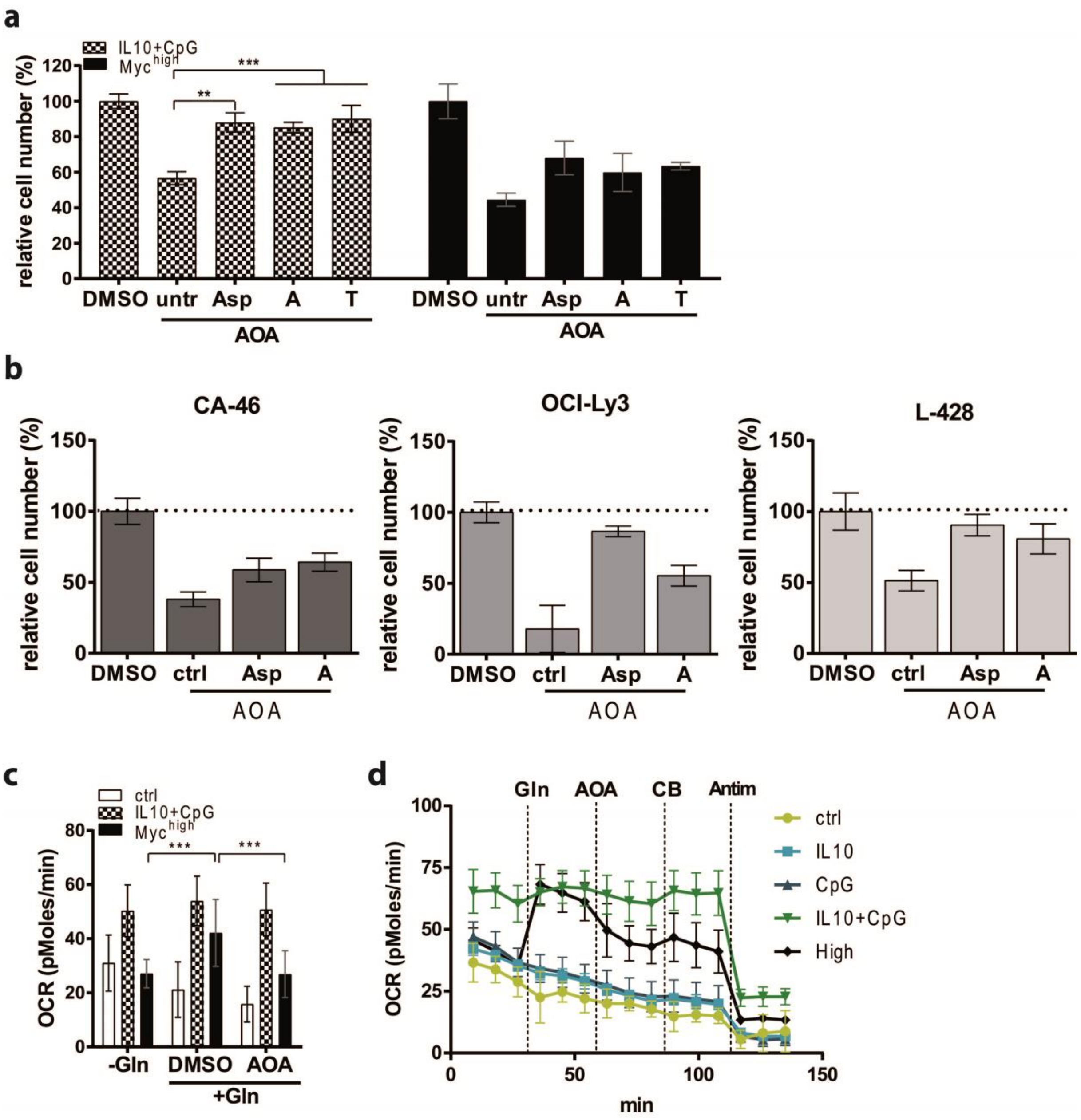

Supplementary Figure 8: The role of glutamine and transaminases in human B-cell lines.

(**a**) Relative cell counts of IL10+CpG-stimulated P493-6 MYC^{low} and unstimulated P493-6 MYC^{high} cells after AOA treatment and indicated Asp, A or T addition. Mean±SD and results from Bonferroni post hoc tests on a one-way ANOVA are shown (* $p<0.05$, ** $p<0.01$, *** $p<0.001$).

(**b**) Relative cell counts of the B-cell lymphoma cells CA-46, OCI-Ly3 and L-428 after AOA treatment and indicated Asp or A addition.

(**c**) OCR of IL10+CpG-stimulated P493-6 MYC^{low} and unstimulated P493-6 MYC^{high} cells seeded in media without Gln. Gln and AOA were added stepwise. One representative analysis out of three is shown. Error bars indicate technical variation. Results of Bonferroni post hoc tests on a one-way ANOVA are depicted (*** $p<0.001$).

(**d**) OCR of IL10+CpG-stimulated P493-6 MYC^{low} and unstimulated P493-6 MYC^{high} cells seeded in media without Gln. Gln, AOA, CB-839, and antimycin were added stepwise. One representative analysis out of three is shown. Error bars indicate technical variation.

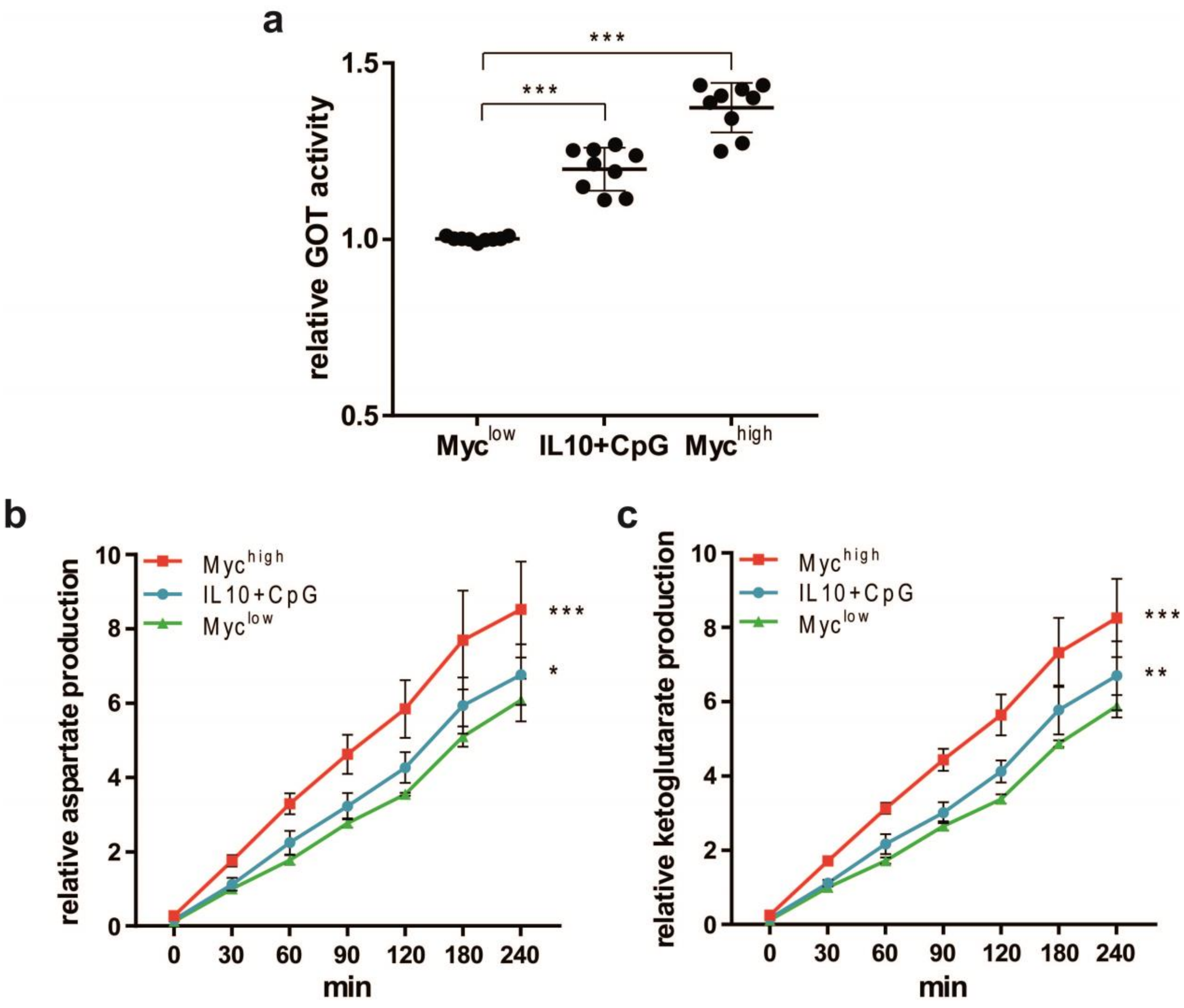

Supplementary Figure 9: GOT activity in IL10+CpG stimulated P493-6 Myc^{low} and unstimulated P493-6 Myc^{high}. (a) Colorimetric measurement of GOT activity. All samples were normalized to unstimulated MYC^{low} cells. Three independent experiments were performed with three technical replicates each. Error bars represent mean±SD and results from Bonferroni post hoc tests on a one-way ANOVA are given (***p<0.001). (**b+c**) Production of (**b**) aspartate and (**c**) alpha-ketoglutarate over time as measured by ^{1}H NMR spectroscopy. Values are given as mean±SD of three independent replicates. Each replicate was adjusted to the 30 min value of the Myc^{low} sample (defined as one). Results from Bonferroni post hoc tests on a two-way ANOVA are presented (***p<0.001, **p<0.01, *p<0.05).

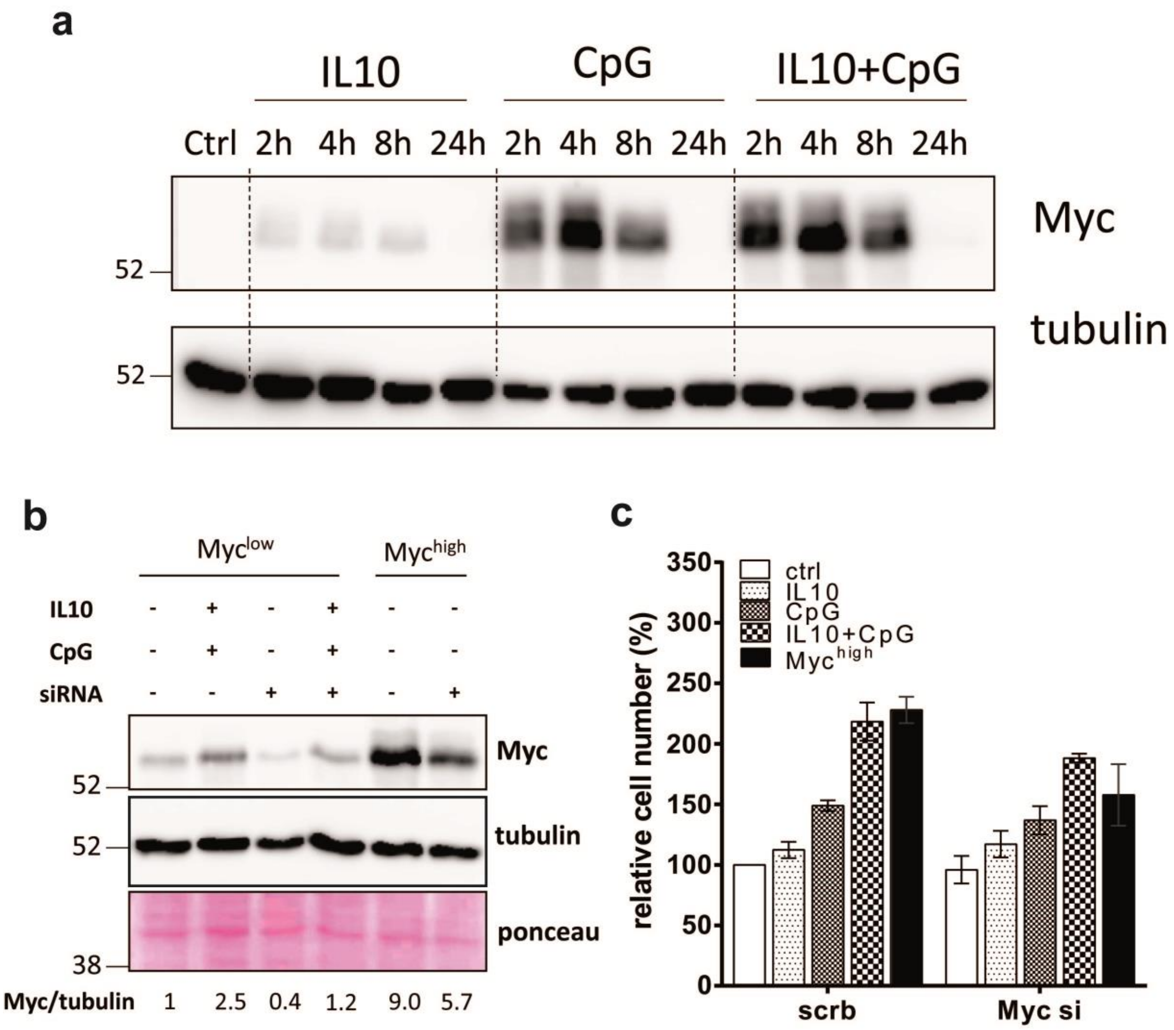

Supplementary Figure 10: Transient increase in Myc protein expression by CpG in P493-6 MYC^{low} cells is insufficient to drive cell proliferation. (**a**) Time dependent changes in Myc protein expression in P493-6 MYC^{low} cells after IL10, CpG, and IL10+CpG stimulation, respectively, as revealed by immunoblotting. (**b**) Immunoblot analysis of Myc protein amounts 24 hours after RNAi mediated MYC knockdown. Shown are Myc, ß-tubulin, and Ponceau red staining, respectively. Indicated are the relative amounts of Myc in relation to tubulin as defined by ImageJ quantification. Please note the relative low knockdown in MYC^{high} cells. (**c**) Cell proliferation of indicated P493-6 cells after RNAi mediated MYC knockdown. Note the greater reduction in cell proliferation of MYC^{high} cells compared to IL10+CpG-stimulated P493-6 MYC^{low} cells despite the about five times larger remaining amount of Myc protein. All analyses were performed in triplicates; representative immunoblots are shown.

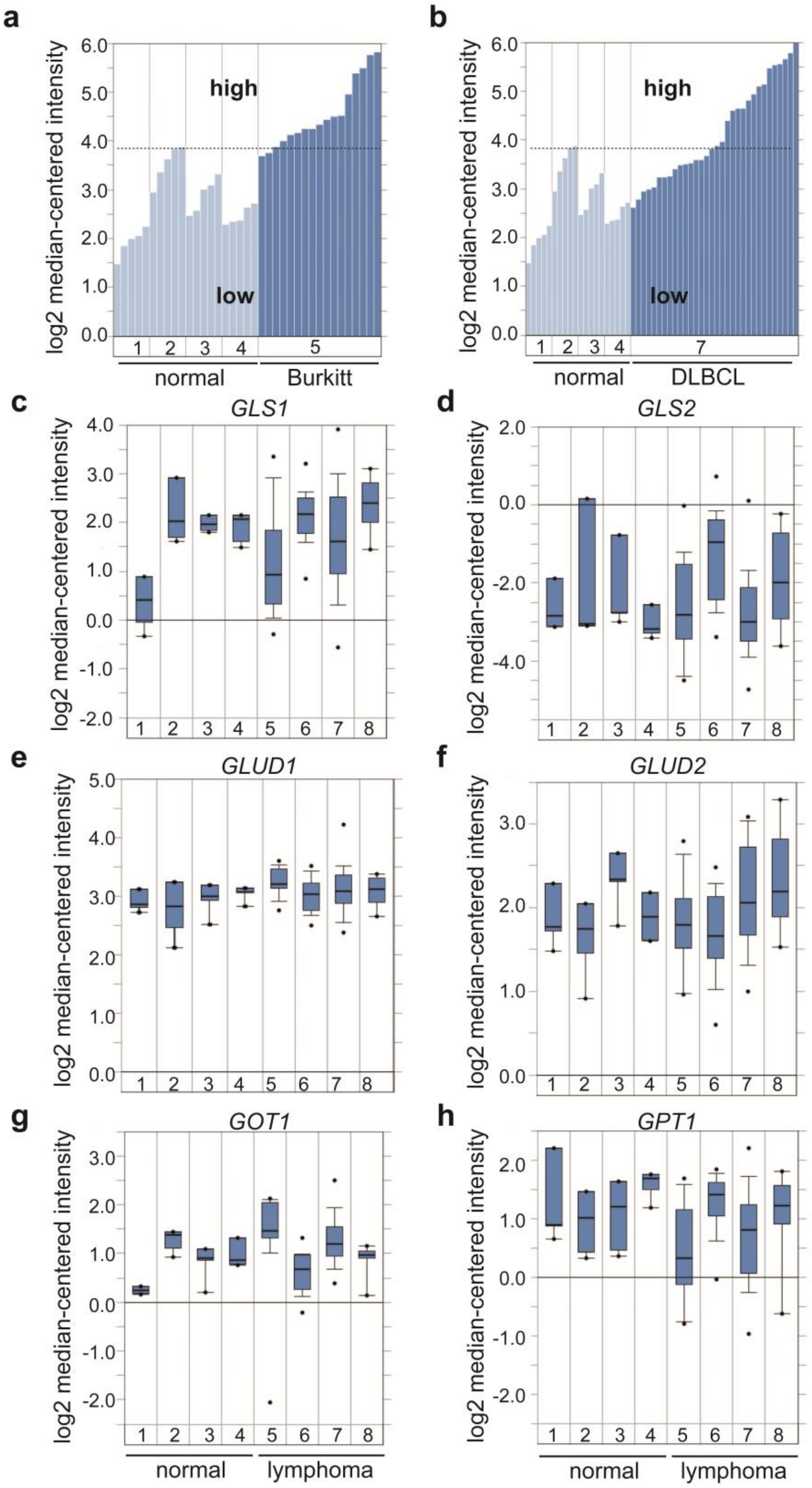

Supplementary Figure 11: *GOT2* is overexpressed in BL and DLBCL, whereas no gene expression differences are observed for other glutaminolysis-related enzymes including *GLS1/2 GLUD1/2, GOT1,* and *GPT1*.

(**a**/**b**) Distribution of *GOT2* expression in BL patients (**a**) and DLBCL patients (**b**) compared to normal B-cell controls (1 = B lymphocyte, 2 = centroblast, 3 = memory B cell, 4 = naive pre-germinal B cell) .

(**c-h**) Gene expression data of glutaminolysis-related genes in normal B cell subsets (1 = B lymphocyte (n=5), 2 = centroblast (n=5), 3 = memory B cell (n=5), 4 = naive pre-germinal B cell (n=5)) and lymphoma samples (5 = BL (n=17), 6 = CLL (n=34), 7 = DLBCL (n=32), 8 = MCL (n=8)). All data were obtained from Basso et al. (2005) as provided on ONCOMINE.

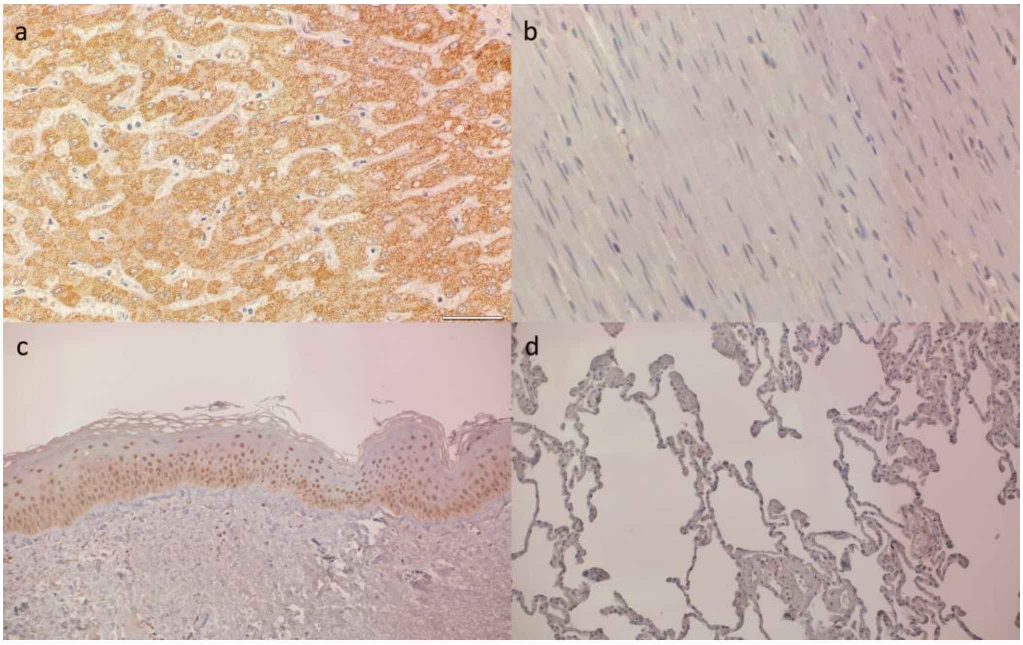

Supplementary Figure 12: Immunohistological staining of GOT2 in representative specimens of (a) liver, (b) muscle, (c) skin, and (d) lung. The bar in (a) is 50 µm. Please note the homogeneous GOT2 staining in the cytosol of hepatocytes. The nuclear staining in skin cells seems to be nonspecific.

Supplementary Table 1: Results from a Cox proportional hazards model to estimate the clinical outcome of patients with DLBCL in relation to the expression of *GOT2*. R-CHOP-treated patients with complete data for all variables (GOT2-GE, age, molecular classification according to ABC-like or GCB-like subtypes of DLBCL and IPI (international prognostic index)) were included (n=157). *GOT2* expression was used as logical parameter (higher median = 1, lower median = 0).

	coef	exp(coef)	se(coef)	z	p-value	significance
**GOT2**	0.70143	2.01663	0.33728	2.08	0.03756	*
**age**	0.0172	1.01735	0.01119	1.537	0.12435	
**ABC**	1.18543	3.27208	0.35899	3.302	0.00096	***
**IPI**	0.77191	2.16389	0.30605	2.522	0.01166	**

Supplementary Table 2: Concentration of stimuli.

Stimulant	Target	Source	Working concentration<br>Full =1	Reduced = 0.2
goat α human IgM $F(ab)_2$	BCR	Jackson ImmunoResearch	1.3 µg/ml	26 ng/ml
ODN2006 (CpG)	TLR9	InvivoGen	0.5 µM	0.1 µM
Recombinant human IGF-1	IGFR	Peprotech	100 ng/ml	20 ng/ml
Recombinant human IL10	IL10R	Peprotech	25 ng/ml	5 ng/ml
sCD40L	CD40	Autogen bioclear	100 ng/ml	20 ng/ml

Supplementary Table 3: Concentration of inhibitors.

Inhibitor	Target	Source	Working concentration
Aminooxyacetic acid (AOA)	Transaminases	Sigma-Aldrich	500 µM
CB-839	GLS	Selleckchem	1 µM
IKK2 inhibitor VIII (ACHP)	IKK2	Calbiochem/Merck	7 µM
Ruxolitinib	JAK1/2	Selleckchem	1 µM

Supplementary Table 4: Concentration of inhibitors.

Metabolite	Source	Working concentration
$^{13}C_5$-Glutamine	Campo Scientific	2 mM
1-^{13}C-glutamine	Campo Scientific	2 mM
alpha-^{15}N glutamine	Campo Scientific	2 mM
Adenine	Sigma-Aldrich	100 µM
Aspartate	Sigma-Aldrich	10 mM
Diethyl malate	Sigma-Aldrich	1 mM
Dimethyl 2-oxoglutarate	Sigma-Aldrich	1 mM
Glucose	Sigma-Aldrich	11 mM
Glutamine	Biochrom/ Merck	2 mM
Oxaloacetic acid	Sigma-Aldrich	1 mM
Pyruvate	Sigma-Aldrich	1 mM
Thymine	Sigma-Aldrich	100 µM

Supplementary Table 5: Matrix of stimuli combinations used for linear regression experiments. P493-6 MYC^{low} (0) and MYC^{high} (1) cells were each stimulated with combinations of α-IgM, CD40L, CpG, IGF-1 or IL10. 1=full concentration, 0.2=reduced concentration, 0=no stimulus added.

Nr.	Batch	CD40L	BCR	IGF	CpG	IL10	MYC	Nr.	Batch	CD40L	BCR	IGF	CpG	IL10	MYC
1	1	1	0	0	0	0	1	51	6	1	0	0	0	0	1
2	1	0	1	0	0	0	0	52	6	0	1	0	0	0	0
3	1	0	0	0	0	0	0	53	6	0	0	0	0	0	0
4	1	0	0	0	0	0	1	54	6	0	0	0	0	0	1
5	1	0	1	1	1	1	1	55	6	0	0	0	0.2	1	1
6	1	1	1	1	1	1	1	56	6	0.2	0.2	0.2	1	1	1
7	1	1	1	0	1	0	1	57	6	0.2	1	1	0	0	1
8	1	1	0	0	1	1	0	58	6	1	1	0	1	1	0
9	1	0.2	1	1	0	1	0	59	6	1	1	1	1	1	0
10	1	0	0	0	1	1	0	60	6	0.2	0	1	0.2	1	0
11	2	0	1	0	0	0	1	61	7	0	1	0	0	0	1
12	2	0	0	1	0	0	0	62	7	0	0	1	0	0	0
13	2	0	0	0	0	0	0	63	7	0	0	0	0	0	0
14	2	0	0	0	0	0	1	64	7	0	0	0	0	0	1
15	2	0	1	0.2	0.2	0	1	65	7	0.2	1	1	0	1	1
16	2	1	0.2	0.2	0	0	1	66	7	1	0.2	0	0	1	1
17	2	1	1	1	1	0.2	1	67	7	1	1	0	1	1	1
18	2	0.2	0.2	0.2	0.2	0.2	0	68	7	0	0.2	0	0.2	1	0
19	2	1	0.2	0	0	1	0	69	7	1	1	1	1	0.2	0
20	2	0.2	1	0.2	0	0.2	0	70	7	1	1	0	1	0	0
21	3	0	0	1	0	0	1	71	8	0	0	1	0	0	1
22	3	0	0	0	1	0	0	72	8	0	0	0	1	0	0
23	3	0	0	0	0	0	0	73	8	0	0	0	0	0	0
24	3	0	0	0	0	0	1	74	8	0	0	0	0	0	1
25	3	0.2	0.2	0.2	0	0	1	75	8	0	0.2	0	0.2	1	1
26	3	0.2	1	0	0	1	1	76	8	0.2	0.2	0.2	0.2	0.2	1
27	3	1	0	0	1	1	1	77	8	0.2	1	0.2	0	0.2	1
28	3	0.2	1	0.2	1	0	0	78	8	0	1	1	1	1	0
29	3	0	0	0	0.2	1	0	79	8	0.2	0.2	0	1	1	0
30	3	0.2	1	0.2	0.2	0.2	0	80	8	0.2	0.2	0.2	1	1	0
31	4	0	0	0	1	0	1	81	9	0	0	0	1	0	1
32	4	0	0	0	0	1	0	82	9	0	0	0	0	1	0
33	4	0	0	0	0	0	0	83	9	0	0	0	0	0	0
34	4	0	0	0	0	0	1	84	9	0	0	0	0	0	1
35	4	0.2	1	0.2	0.2	0.2	1	85	9	0.2	1	1	0.2	0	1
36	4	1	1	0.2	0.2	0	1	86	9	0.2	0.2	1	0	0.2	1
37	4	0.2	0	1	0.2	0	1	87	9	1	0	1	1	0.2	1
38	4	1	1	0.2	0.2	0	0	88	9	0	1	0.2	0.2	0	0
39	4	0.2	1	1	0.2	0	0	89	9	0.2	0.2	1	0	0.2	0
40	4	1	0	1	1	0.2	0	90	9	1	0	1	0.2	0	0
41	5	0	0	0	0	1	1	91	10	0	0	0	0	1	1
42	5	1	0	0	0	0	0	92	10	1	0	0	0	0	0
43	5	0	0	0	0	0	0	93	10	0	0	0	0	0	0
44	5	0	0	0	0	0	1	94	10	0	0	0	0	0	1
45	5	0	0	0	1	1	1	95	10	1	0	1	0.2	0	1
46	5	0.2	0.2	0	1	1	1	96	10	0.2	1	0.2	1	0	1
47	5	0.2	0	0.2	1	0	1	97	10	0.2	0	1	0.2	1	1
48	5	0.2	0.2	0.2	0	0	0	98	10	0.2	1	1	0	0	0
49	5	0.2	1	0	0	1	0	99	10	0.2	0	1	0.2	0	0
50	5	0.2	0	0.2	1	0	0	100	10	1	0.2	0.2	0	0	0

Supplementary Table 6: siRNA sequences. For all transfections ON-TARGETplus Human siRNA-SMARTpools from Dharmacon were used. Sequences according to corresponding data sheets.

GOT1 SMARTpool	GAGCAUAUCGCACGGAUGA
	UAGCCUAAAUCACGAGUAU
	GAACAGGUGCACUUCGAAU
	GAGAAGAUCGUGCGGAUUA
GOT2 SMARTpool	GCUACAAGGUUAUCGGUAU
	CUUGAAGAGUGGCCGGUUU
	GCUUAUAUGGUGAGCGUGU
	UGAGAAACAGCACACGUUA
GPT2 SMARTpool	AUGAGCAGGUGUCGGGAAA
	GUGAAAAGGUUAAAUCGUA
	AGAAGUGCUGUACGAGAU
	GAGCAAUUCAGCCGAGAGA
STAT3 SMARTpool	GAGAUUGACCAGCAGUAUA
	CAACAUGUCAUUUGCUGAA
	CCAACAAUCCCAAGAAUGU
	CAACAGAUUGCCUGCAUUG
RELA SMARTpool	GGAUUGAGGAGAAACGUAA
	CCCACGAGCUUGUAGGAAA
	GGCUAUAACUCGCCUAGUG
	CCACACAACUGAGCCCAUG

Supplementary Table 7: Cell line specific transfection parameters. Shown are parameters for a single transfection.

**Cell line**	**Number of cells**	**siRNA**	**Kit**	**program**
P493-6	$4 \cdot 10^6$	4 µg	V	C-009
OCI-Ly3	$2 \cdot 10^6$	2 µg	V	A-020
L-428	$2 \cdot 10^6$	2 µg	L	X-001
CA-46	$2 \cdot 10^6$	2 µg	V	R-013

Supplementary Table 8: Port loading for respiration analysis in Supplementary Figure 6.

Port	Substance	Concentration
A	Oligomycine	1.5 µM
B	FCCP	0.5 µM
C	Rotenone	2 µM
D	Antimycin A	1 µM

Supplementary Table 9 : Port loading for respiration analysis in Supplementary Figure 8C,D.

Port	Substance	Concentration
A	Gln	2 mM
B	AOA	500 µM
C	CB-839	1 µM
D	Antimycin A	1µM

Supplementary Table 10: concentrations used in Supplementary Figure 6C.

Substance	Concentration
UK5099	3µM
BPTES	4µM
etomoxir	5µM

Supplementary Table 11: Measuring protocol

Command	Time(min)	Port	Repeats
Calibrate			
Equilibrate			
Mix	2		
Wait	2		
Measure	4		
Inject		A	
Mix	2		
Wait	2		3
Measure	4		
Inject		B	
Mix	2		
Wait	2		3
Measure	4		
Inject		C	
Mix	2		
Wait	2		3
Measure	4		
Inject		D	
Mix	2		
Wait	2		3
Measure	4		

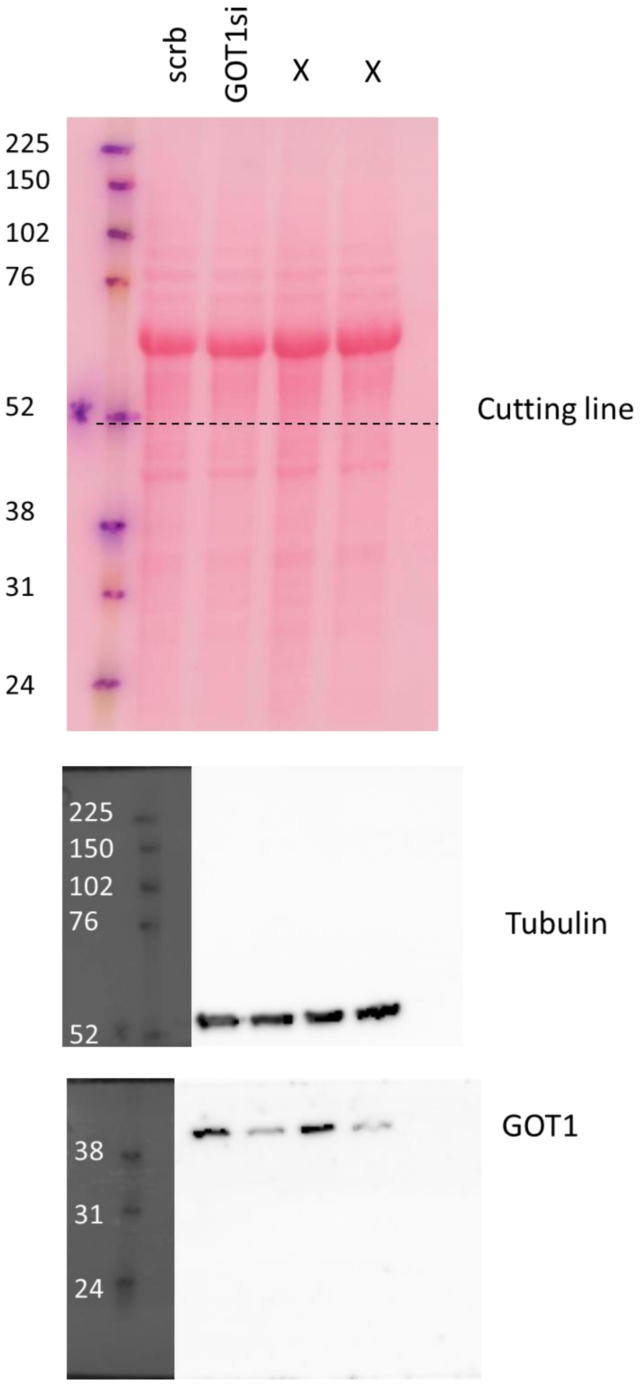

Supplementary Figure 13: Uncropped version of GOT1 western blot shown in Figure 4.
The first two lanes (scrb, GOT1si) were used for Figure 4a. Equal loading was controlled by ponceau staining. Membrane was cut in half prior to staining of tubulin and GOT1. Size marker (kDa) is shown on the left side.

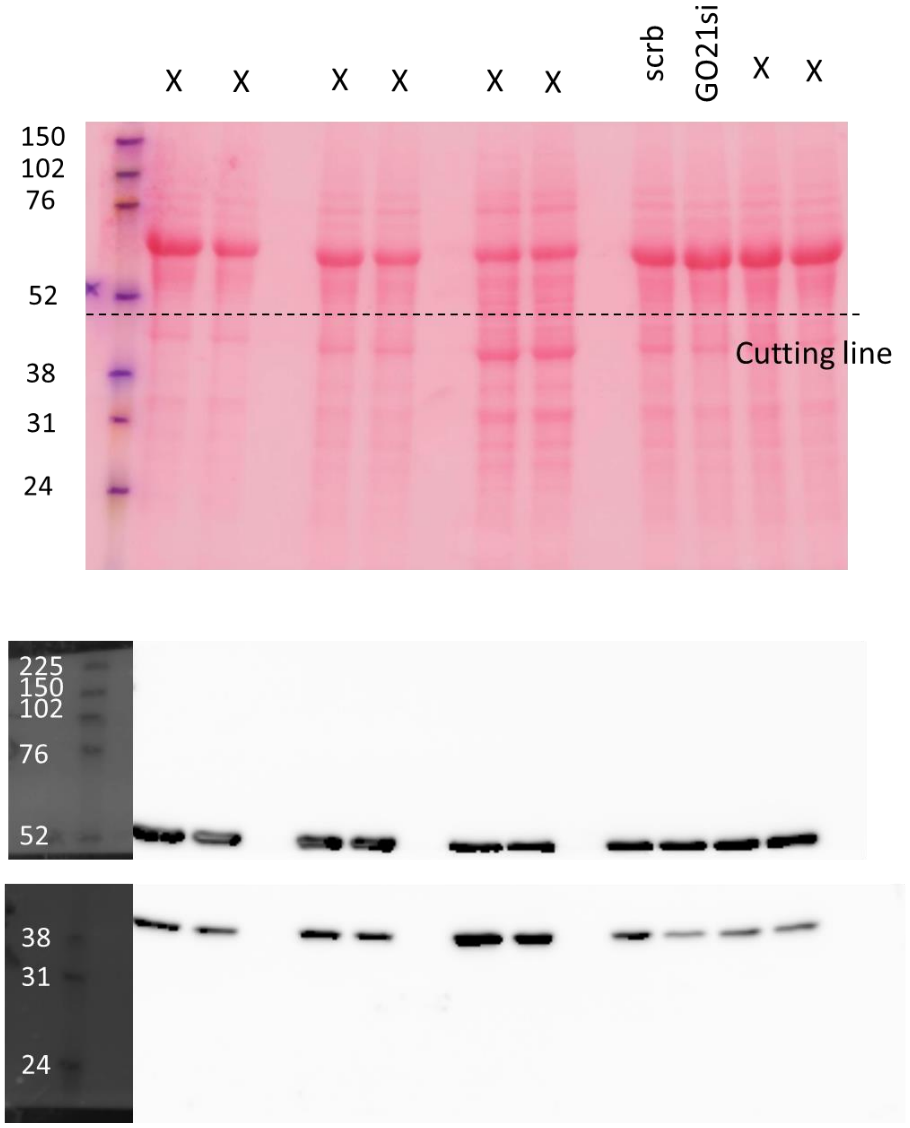

Supplementary Figure 14: Uncropped version of GOT2 western blot shown in Figure 4.

Lane 7 and 8 (scrb, GOT2si) were used for Figure 4a. Equal loading was controlled by ponceau staining. Membrane was cut in half prior to staining of tubulin and GOT2. Size marker (kDa) is shown on the left side.

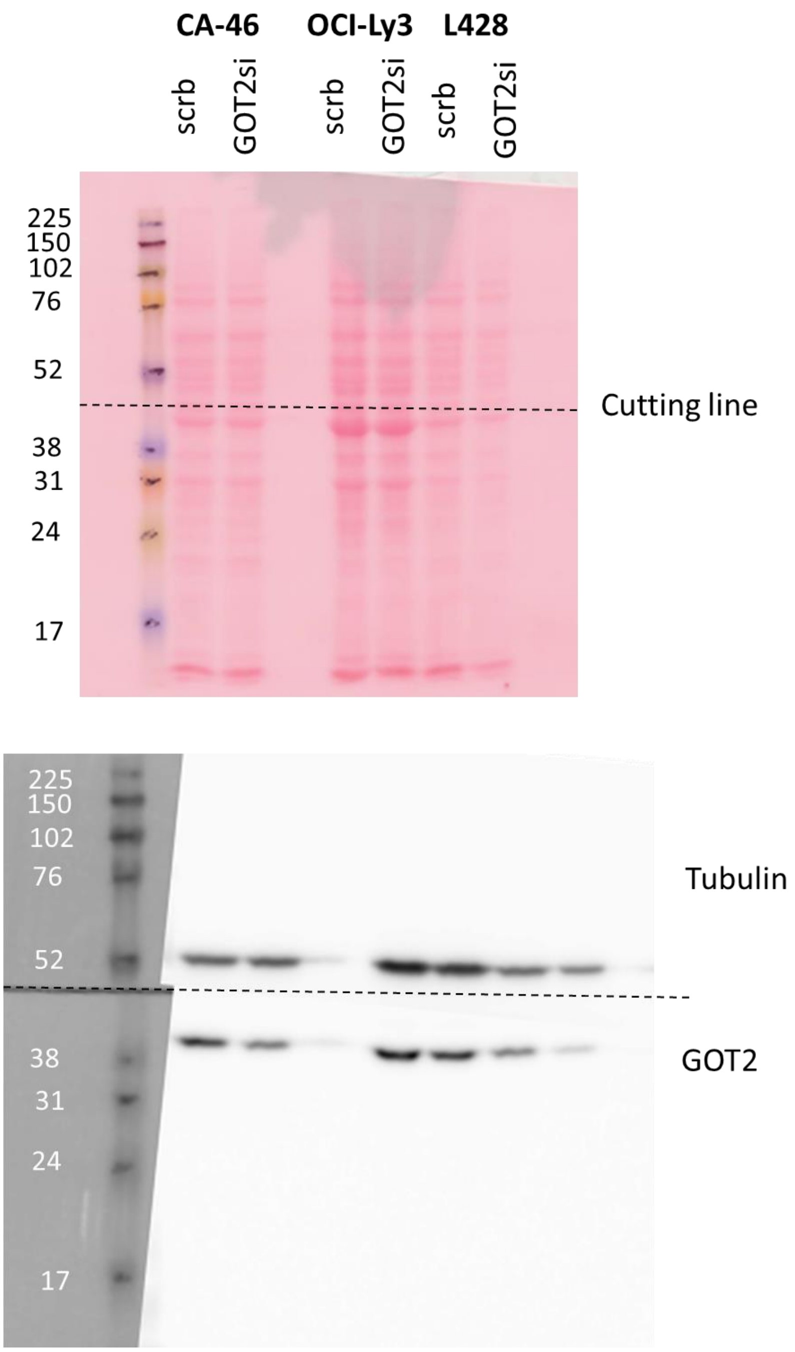

Supplementary Figure 15: Uncropped version of GOT2 western blot shown in Figure 5.
Equal loading was controlled by ponceau staining. Membrane was cut in half prior to staining of tubulin and GOT2. Size marker (kDa) is shown on the left side.

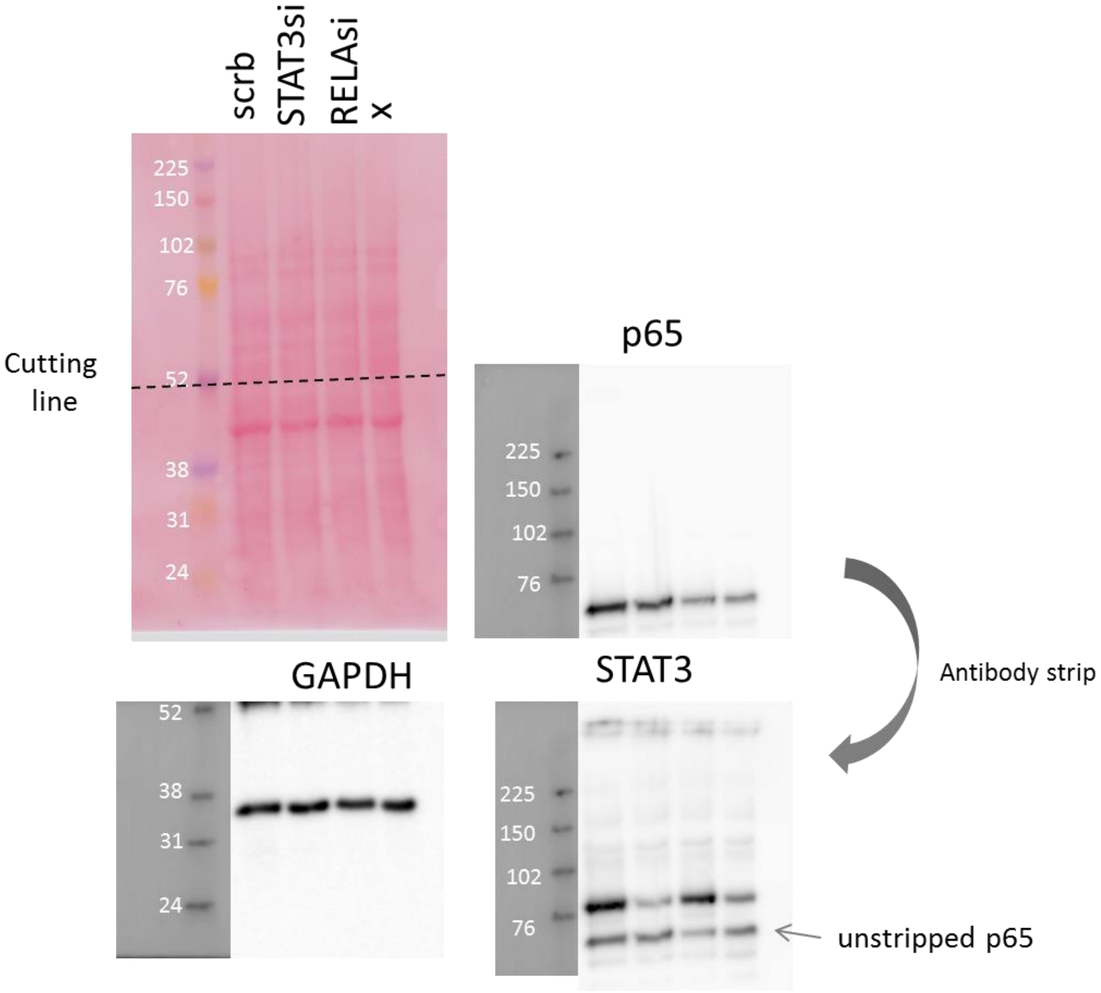

Supplementary Figure 16: Uncropped version of the western blot shown in Figure 6.

First three lanes were used for Figure 6b. Equal loading was controlled by ponceau staining. Membrane was cut in half prior to staining of p65 and GAPDH. Upper membrane part was stripped and stained for STAT3. Size marker (kDa) is shown on the left side.

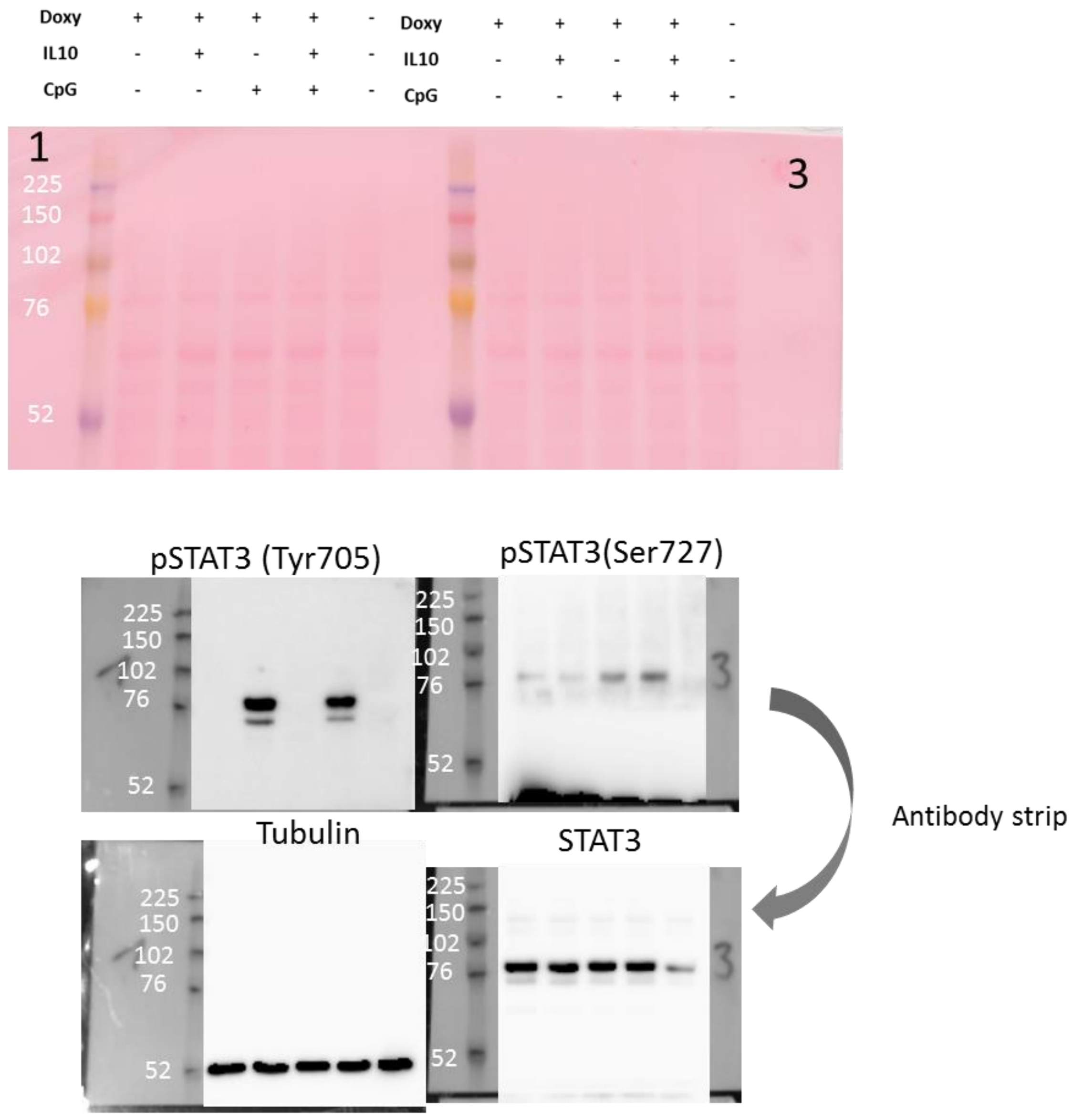

Supplementary Figure 17: Uncropped version of the western blot shown in Figure 6e. Samples were loaded twice on the same gel. Membrane was cut in half. Numbers indicate which membrane part was used. Equal loading was shown by ponceau staining. Tubulin and total STAT3 were stained after stripping. Size marker (kDa) is shown on the left side

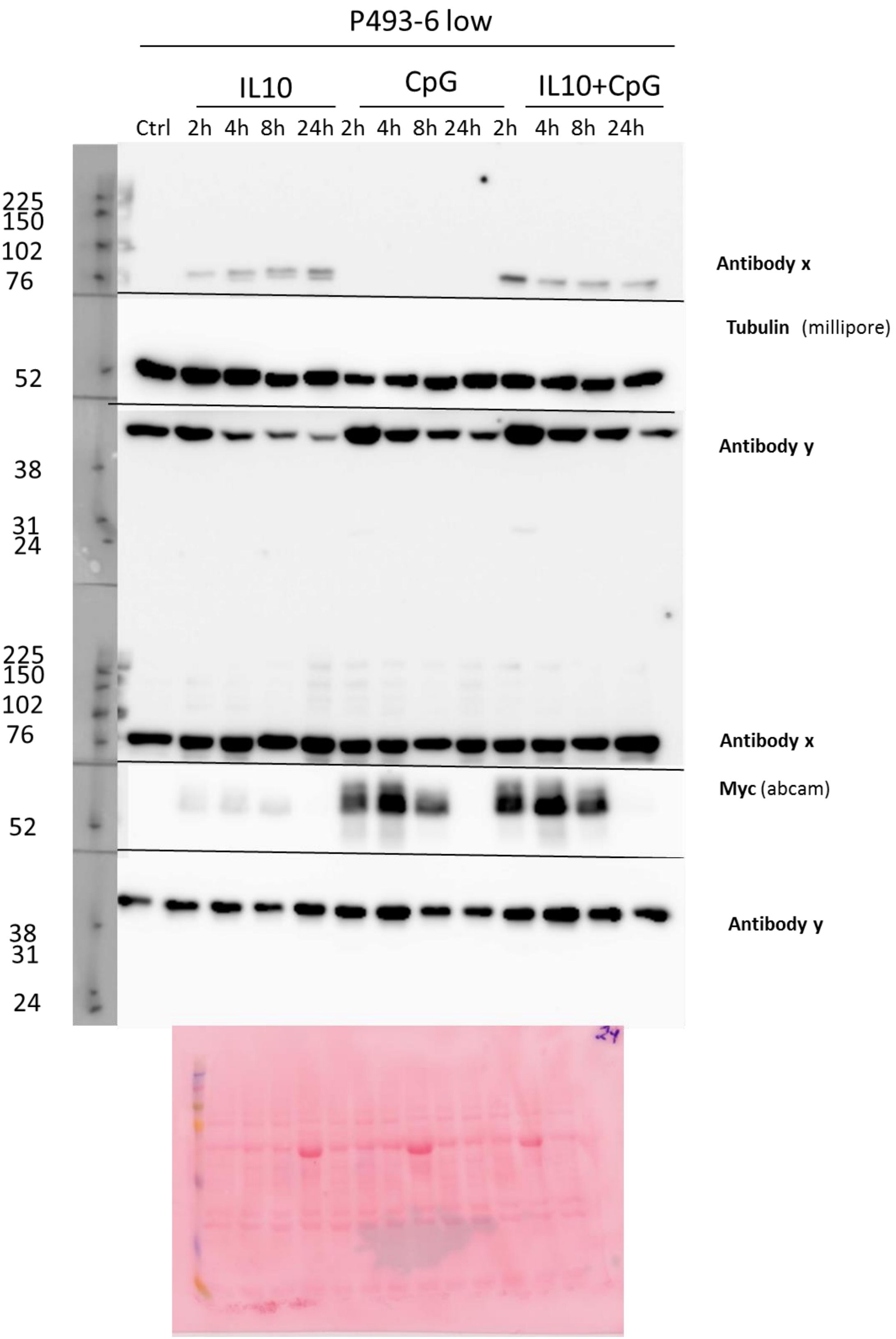

Supplementary Figure 18: Uncropped version of the western blot shown in Supplementary Figure 10a. Membrane was cut in three parts. Equal loading was shown by ponceau staining. Size marker (kDa) is shown on the left side

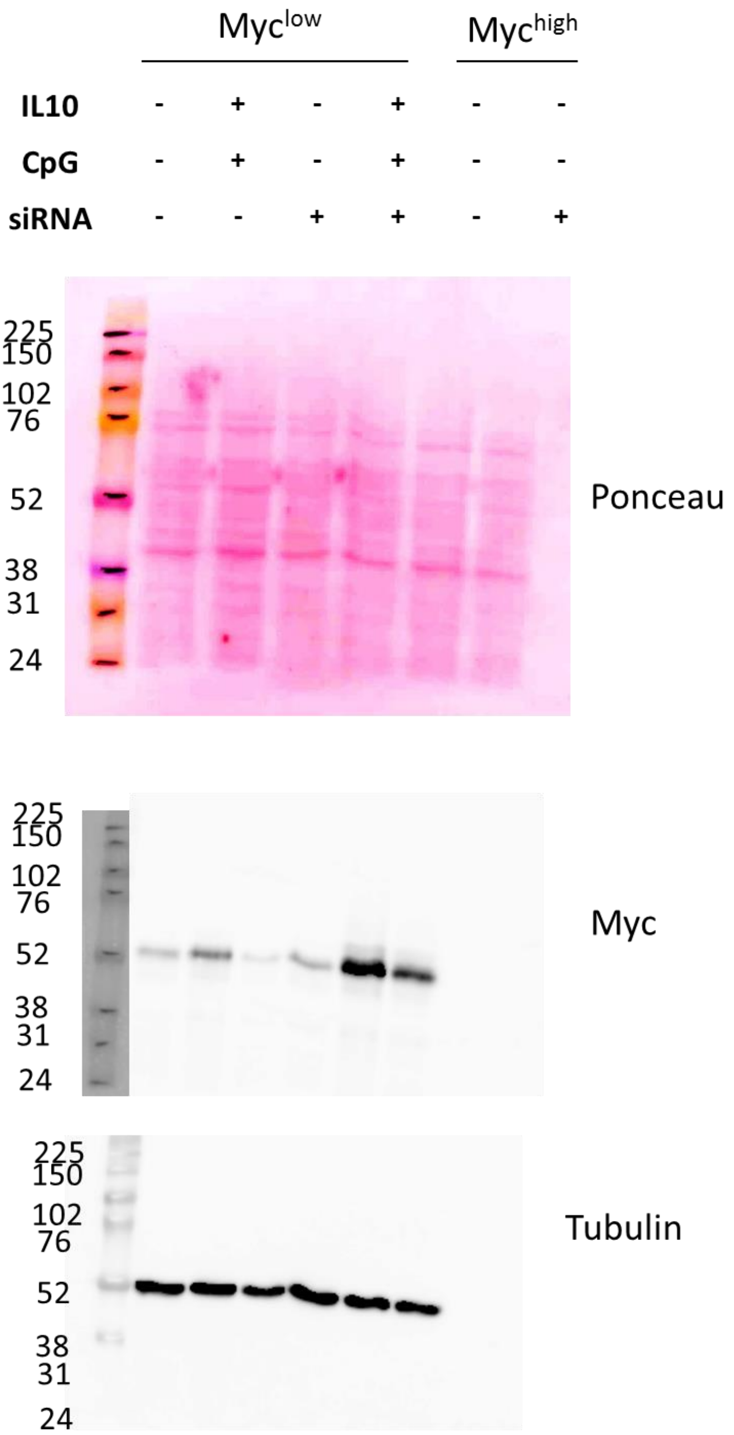

Supplementary Figure 19: Uncropped version of the western blot shown in Supplementary Figure 10b. Equal loading was shown by ponceau staining. Tubulin was stained after stripping. Size marker (kDa) is shown on the left side
