# Supplementary material for: Cooperative STAT/NF-κB signaling regulates lymphoma metabolic reprogramming and aberrant GOT2 expression
Source: Nat Commun. 2018 Apr 17;9:1514. doi: 10.1038/s41467-018-03803-x (PMC5904148; doi:10.1038/s41467-018-03803-x)
Supplement: Supplementary file 3 — Description of Additional Supplementary Files [file 41467_2018_3803_MOESM3_ESM.pdf]

Description of Additional Supplementary Files

File Name: Supplementary Data 1
Description: Changes in protein abundance as determined by targeted proteomics (SWATH sequential window acquisition of all theoretical fragment ion spectra).
